# The predictive value of the Oxford Acute Severity of Illness Score for clinical outcomes in patients with acute kidney injury

**DOI:** 10.1080/0886022X.2022.2027247

**Published:** 2022-02-16

**Authors:** Na Wang, Meiping Wang, Li Jiang, Bin Du, Bo Zhu, Xiuming Xi

**Affiliations:** a Emergency Department of China Rehabilitation Research Center, Capital Medical University, Beijing, China; b Department of Epidemiology and Health Statistics, School of Public Health, Capital Medical University, Beijing, China; c Department of Critical Care Medicine, Xuan Wu Hospital, Capital Medical University, Beijing, China; d Medical Intensive Care Unit, Peking Union Medical College Hospital, Peking Union Medical College and Chinese Academy of Medical Sciences, Beijing, China; e Department of Critical Care Medicine, Fu Xing Hospital, Capital Medical University, Beijing, China

**Keywords:** Oxford Acute Severity of Illness Score, intensive care unit, AKI, mortality

## Abstract

**Objective:**

To compare the performance of the Oxford Acute Severity of Illness Score (OASIS), the Acute Physiology and Chronic Health Evaluation II (APACHE II) score, the Simplified Acute Physiology Score II (SAPS II), and the Sequential Organ Failure Assessment (SOFA) score in predicting 28-day mortality in acute kidney injury (AKI) patients.

**Methods:**

Data were extracted from the Beijing Acute Kidney Injury Trial (BAKIT). A total of 2954 patients with complete clinical data were included in this study. Receiver operating characteristic (ROC) curves were used to analyze and evaluate the predictive effects of the four scoring systems on the 28-day mortality risk of AKI patients and each subgroup. The best cutoff value was identified by the highest combined sensitivity and specificity using Youden’s index.

**Results:**

Among the four scoring systems, the area under the curve (AUC) of OASIS was the highest. The comparison of AUC values of different scoring systems showed that there were no significant differences among OASIS, APACHE II, and SAPS II, which were better than SOFA. Moreover, logistic analysis revealed that OASIS was an independent risk factor for 28-day mortality in AKI patients. OASIS also had good predictive ability for the 28-day mortality of each subgroup of AKI patients.

**Conclusion:**

OASIS, APACHE II, and SAPS II all presented good discrimination and calibration in predicting the 28-day mortality risk of AKI patients. OASIS, APACHE II, and SAPS II had better predictive accuracy than SOFA, but due to the complexity of APACHE II and SAPS II calculations, OASIS is a good substitute.

**Trial Registration:**

This study was registered at www.chictr.org.cn (registration number Chi CTR-ONC-11001875). Registered on 14 December 2011.

## Introduction

Acute kidney injury (AKI) is a common and serious complication in intensive care unit (ICU) patients, and it is an important risk factor for increased early and long-term morbidity and mortality during hospitalization [1–4]. Early identification and diagnosis, correct assessment of prognosis, and active treatment are the keys to reducing the mortality rate. Many severity scores have been developed to evaluate the prognosis of the critically ill patients, including AKI patients.

The Acute Physiology and Chronic Health Evaluation II (APACHE II) score is the most commonly used disease severity scoring system in ICUs around the world [5]; it includes 12 physiological and laboratory parameters and two disease-related variables [6]. The Simplified Acute Physiology Score II (SAPS II) was first described in 1984 as an alternative to the APACHE scoring system [7], and it is an effective tool for evaluating AKI patient outcomes [8,9]. However, all of the above models require considerable effort for data collection. Although the Sequential Organ Failure Assessment (SOFA) score [10] is simple to use and accurate in predicting the mortality outcome of AKI patients [11–13], it depends on laboratory results, and some important prognostic factors were not included.

In 2013, Johnson et al. performed a retrospective cohort study of 72 474 ICU patients in 68 ICUs at 49 U.S. hospitals from 2007 to 2011 and developed a new reduced severity of illness score using machine learning algorithms, the Oxford Acute Severity of Illness Score (OASIS), which contained 10 parameters without any laboratory tests and had discrimination and calibration equivalent to more complex existing models, the highest score is 75 [14].

The predictive value of OASIS was validated in mixed ICU patient populations, but its performance in AKI patients remains unknown. The aim of this multicenter study was to evaluate the performance of OASIS for the assessment of mortality in AKI patients in China, and compare with APACHE II, SAPS II, and SOFA.

## Methods

### Study setting and data collection

This study used a database from the Beijing Acute Kidney Injury Trial (BAKIT) [15], a prospective, multicenter, observational study that investigated the epidemiology of acute kidney injury (AKI) in critically ill patients in 30 ICUs at 28 tertiary hospitals in Beijing, China, conducted between 1 March and 31 August 2012 (for a complete list of these hospitals and the persons responsible for the data acquisition, see Additional file 1). The study subjects included all adult patients (age ≥ 18 years) admitted consecutively to the ICUs. Only the initial ICU admission was considered in this study. The following patients were excluded: patients with preexisting end-stage chronic kidney disease, patients already receiving renal replacement therapy (RRT) before admission to the ICU, and patients who had received kidney transplantation in the previous three months. Preexisting comorbidities were diagnosed based on the International Classification of Diseases (ICD-10) codes. The patients were followed up until death, until hospital discharge, or for 28 days.

Thorough follow-up of all patients included in the study was conducted in the first 10 days after ICU admission. The collected data included demographics, anthropometrics, admission diagnosis, comorbidities, daily vital signs and laboratory data (which were used to automatically calculate the APACHE II score, the SAPS II, and the SOFA score), days from hospital to ICU admission, ICU length of stay (LOS), hospital LOS, use of vasoactive drugs, the occurrence of AKI, and length of mechanical ventilation (MV). RRT data were also reported.

Mortality data up to 28 days after ICU discharge were collected from hospital records, including records from hospital admissions and visits to outpatient clinics.

AKI was defined and classified according to the Kidney Disease Improving Global Outcomes (KDIGO) guidelines [16]. Patients were categorized on the basis of serum creatinine (SCr) or urine output or both. Baseline creatinine was defined as the lowest known SCr value in the last three months [17]. For patients without baseline creatinine, we used the estimated baseline creatinine or the lowest SCr in the ICU course, whichever was lower. The baseline creatinine was estimated by the Modification of Diet in Renal Disease (MDRD) equation [18], assuming a glomerular filtration rate of 75 mL/min/1.73m^2^ [19].

We calculated the OASIS within the first day of ICU admission. The parameters used to calculate the OASIS are shown in Table S1.

### Outcomes

The primary outcome was 28-day mortality, and the secondary outcomes were ICU mortality and hospital mortality. The ICU LOS and hospital LOS were calculated only for statistical description. ICU mortality and ICU LOS were determined by the first ICU stay only.

### Statistical analysis

Nonnormally distributed continuous variables are expressed as the medians with interquartile ranges (IQRs) and were compared using the Mann-Whitney U test or Kruskal-Wallis analysis-of-variance test with Bonferroni correction. Categorical variables are expressed as the number of cases and proportions and were compared using the Mantel-Haenszel Chi-square test.

Receiver operating characteristic (ROC) curves were drawn according to the sensitivity and specificity of the four scoring systems for predicting the 28-day mortality risk of patients. The ROC curve comparison function of Medcalc software was used for pairwise comparisons of the area under the curve (AUC), the larger the AUC, the higher the predictive value. AUCs of ≥ 0.9, 0.8 to 0.89, 0.7 to 0.79, 0.6 to 0.69 or < 0.6 were classified as excellent, very good, good, fair, and poor, respectively.

Cutoff values, sensitivities, specificities, positive predictive values, and negative predictive values were calculated by ROC analysis. The best cutoff values for the prediction of 28-day mortality, ICU mortality, and hospital mortality were determined by the maximum of the Youden index (i.e., sensitivity plus specificity minus one) calculated from the ROC analysis. The Hosmer- Lemeshow goodness-of-fit test was used to test the calibration of the scoring system.

We used a logistic regression model to evaluate the effect of OASIS on the 28-day mortality in AKI patients. Because OASIS was collinear with APACHE II and SAPS II scores, the variables considered for multivariable analysis included age, sex, OASIS, SOFA, use of vasoactive drugs, MV, RRT, and underlying diseases. OASIS was entered as a continuous variable and a categorical variable, respectively.

To verify the predictive effect of OASIS on the 28-day mortality of patients with different AKI grades and among different populations of AKI patients, subgroup analyses were performed by ROC analysis.

All statistical analyses were performed using SPSS software (IBM Corp., Statistics for Windows, version 22.0, Armonk, NY, USA), with a two-sided *p* values < .05 considered statistically significant.

## Results

### Study population

**Figure 1.**
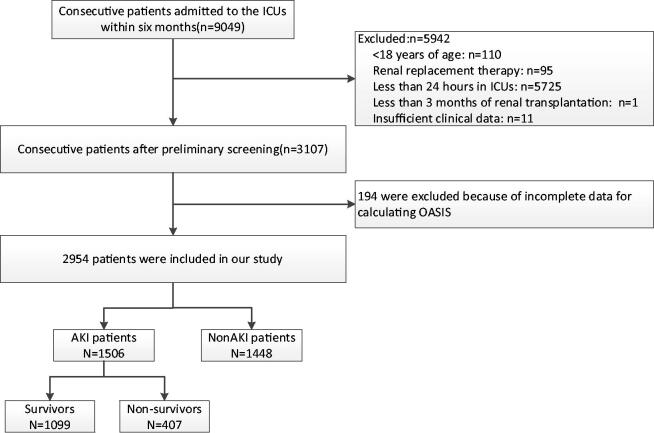
Study flow chart with 28-day mortality rate.

During the study period, 9079 patients were admitted consecutively. Of them, patients were excluded because of the following reasons: 5725 patients had an ICU LOS of less than 24 h, 110 patients were younger than 18 years old, one patient received renal transplantation during the past three months, 95 patients had received RRT before admission to the ICU, and 11 had insufficient clinical recordings. Thus, 3107 patients were enrolled in the BAKIT study. Of these patients, 194 were excluded because of incomplete data for calculating OASIS, and finally, 2954 patients were included in our study (Figure 1).

The characteristics of the entire cohort are shown in Table 1. The median age was 64 (IQR: 51–76) years, and 61.6% were men. The all-cause 28-day mortality rate was 17.0%, and the median ICU LOS was 4 (IQR: 2–9) days. Among the included patients, the median OASIS was 28 (IQR: 23–36), the median APACHE II score was 14 (IQR: 10–20), the median SAPS II was 6 (IQR: 3–8), and the median SOFA score was 6 (IQR: 3–8). MV was used in 1960 (66.4%) patients, 1230 patients (41.6%) received vasopressors, 1506 (51.0%) patients developed AKI as defined by the KDIGO criteria, and 252 patients (8.5%) underwent RRT.

**Table 1.** Patient characteristics by 28-day mortality.

Characteristic	All patients (*n* = 2954) Median (IQR) Number (%)	Survivors (*n* = 2453) Median (IQR) Number (%)	Non-survivors (*n* = 501) Median (IQR) Number (%)	*p*
Age (years)	64 (51–76)	63 (50–75)	72 (59–81)	<.001
Male sex	1819 (61.6)	1518 (61.9)	301 (60.1)	.751
**ICU course**				
Vasoactive therapy	1230 (41.6)	1033 (42.1)	197 (39.3)	.457
MV	1960 (66.4)	1591 (64.9)	369 (73.7)	<.001
Sepsis	848 (28.7)	540 (22.0)	308 (61.5)	<.001
AKI	1506 (51.0)	1099 (44.8)	407 (81.2)	<.001
RRT	252 (8.5)	127 (5.2)	125 (25.0)	<.001
**Severity of illness**				
OASIS	28 (23–36)	27 (22–33)	38 (31–45)	<.001
APACHEII	14 (10–20)	13 (9–18)	22 (17–28)	<.001
SAPSII	33 (25–44)	31 (24–40)	50 (39–63)	<.001
SOFA	6 (3–8)	5 (3–7)	9 (6–11)	<.001
**Admission category**				
Emergency	1068 (36.2)	732 (29.8)	336 (67.1)	<.001
Urgent	427 (14.5)	367 (15.0)	60 (12.0)	
Elective	1459 (49.4)	1354 (53.2)	105 (20.9)	
**Comorbidities**				
Hypertension	1176 (39.8)	949 (37.3)	227 (45.3)	
Coronary heart disease	569 (19.3)	427 (17.4)	142 (28.3)	
Congestive heart failure	188 (6.4)	113 (4.6)	75 (15.0)	
COPD	158 (5.3)	120 (4.9)	38 (7.6)	
Diabetes	511 (17.3)	418 (17.0)	93 (18.6)	
Chronic kidney disease	151 (5.1)	108 (4.4)	43 (8.6)	
Liver disease	82 (2.8)	63 (2.6)	19 (3.8)	
Cancer	407 (13.8)	352 (14.3)	55 (11.0)	
Hematological disease	24 (0.8)	13 (0.5)	11 (2.2)	
**Category of ICU admission diagnosis**				
Cardiovascular	820 (27.8)	733 (28.8)	87 (17.4)	
Respiratory	516 (17.5)	356 (14.0)	160 (31.9)	
Neurologic	436 (14.8)	337 (13.3)	99 (19.8)	
Trauma	225 (7.6)	203 (8.0)	22 (4.4)	
Gastrointestinal	578 (19.6)	485 (19.1)	93 (18.6)	
Metabolic	66 (2.2)	54 (2.1)	12 (2.4)	
**Outcomes**				
ICU LOS (days)	4 (2–9)	4 (2–7)	6 (3–13)	<.001
Hospital LOS (days)	19 (12–29)	19 (12–28)	21 (11–34)	.002

Data are expressed as the median (interquartile range, IQR), and number (percentage).

MV: mechanical ventilation; AKI: acute kidney injury; RRT: renal replacement therapy; the Oxford Acute Severity of Illness Score; APACHE II: Acute Physiology and Chronic Health Evaluation II; SAPS II: Simplified Acute Physiology Score II; SOFA: Sequential Organ Failure Assessment; COPD: chronic obstructive pulmonary disease; LOS: length of stay.

There were statistically significant differences in age, MV, sepsis, AKI, RRT, OASIS, APACHE II, SAPS II, SOFA, admission category, ICU LOS, and hospital LOS between survivors and non-survivors.

### Comparison of characteristics between the survival and non-survival groups of AKI patients

AKI patient characteristics according to 28-day mortality are shown in Table 2. Non-surviving AKI patients were older (*p* < .001), had higher illness severity scores, and were more likely to be diagnosed with sepsis. Positive fluid balance in the first 24 h was more common among non-survivors.

**Table 2.** AKI patient characteristics by 28-day mortality.

Characteristic	AKI patients (*n* = 1506) Median (IQR) Number (%)	Survivors (*n* = 1099) Median (IQR) Number (%)	Non-survivors (*n* = 407) Median (IQR) Number (%)	*p*
Age (years)	67 (53–78)	64 (51–77)	74 (59–81)	<.001
Male gender	918 (61.0)	674 (61.3)	244 (60.0)	.886
Baseline creatinine (µmol/L)	84.0 (71.6–97.0)	83.4 (71.0–97.0)	85.0 (75.0–97.3)	.685
**Severity of illness**				
APACHEII	17 (12–23)	15 (10–20)	23 (18–29)	<.001
SAPSII	39 (30–52)	35 (27–45)	52 (41–65)	<.001
SOFA	7 (4–10)	6 (4–9)	9 (6–12)	<.001
OASIS	31 (24–39)	28 (23–35)	39 (32–46)	<.001
**ICU course**				
Vasoactive therapy	622 (41.3)	451 (41.0)	171 (42.0)	.942
MV	1052 (69.9)	748 (68.1)	304 (74.7)	.045
Sepsis	603 (40.0)	335 (30.5)	268 (65.8)	<.001
Positive fluid balance first 24 hours	1083 (71.9)	743 (67.6)	340 (83.5)	<.001
Use of diuretics on the first day of admission	510 (33.9)	368 (33.5)	142 (34.9)	.876
Staging of AKI				
1	699 (46.4)	592 (53.9)	107 (26.3)	
2	357 (23.7)	260 (23.7)	97 (23.8)	<.001
3	450 (29.9)	247 (22.5)	203 (49.9)	
RRT	241 (16.0)	121 (11.0)	120 (29.5)	<.001
**Outcomes**				
Hospital LOS (days)	20 (11–30)	22 (14–34)	13 (6–23)	<.001
ICU LOS (days)	5 (3–11)	5 (3–11)	6 (4–12)	.030

Data are expressed as the median (interquartile range, IQR), and number (percentage).

AKI: acute kidney injury; the Oxford Acute Severity of Illness Score; SAPS II: Simplified Acute Physiology Score II; SOFA: Sequential Organ Failure Assessment; APACHE II: Acute Physiology and Chronic Health Evaluation II; MV; mechanical ventilation; LOS: length of stay; RRT: renal replacement therapy.

### The 28-day mortality of AKI patients according to OASIS

**Figure 2.**
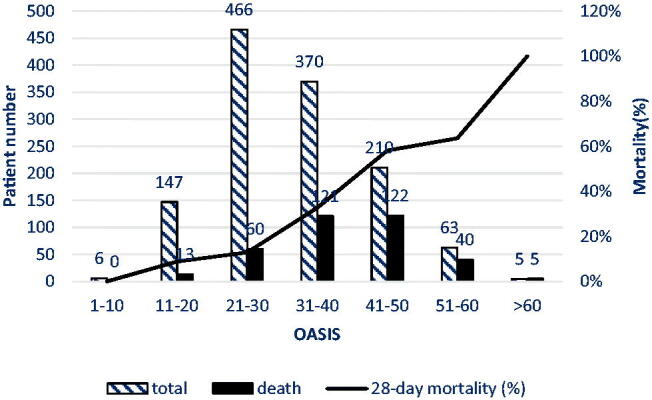
The 28-day mortality in AKI patients according to OASIS.

The distribution of OASIS in AKI patients is shown in Figure 2. OASIS ranged from 6 to 64, and the median value was 31 (IQR: 24, 39). The distributions of the OASIS with corresponding 28-day mortality are also presented in Figure 2. As each score increased, the 28-day mortality of AKI patients increased accordingly, indicating more serious illness and worse prognosis.

### Comparison of ROC curve and AUCs of the four scoring systems in evaluating the 28-day mortality of AKI patients

**Figure 3.**
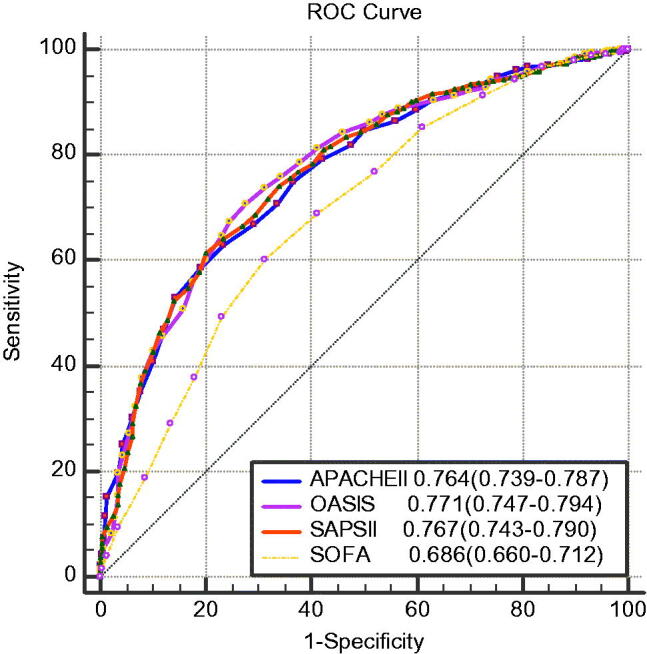
ROC curves of OASIS, APACHE II, SAPS II, and SOFA score for 28-day mortality in AKI patients.

In Figure 3, OASIS had the highest discriminatory power in predicting the prognosis of AKI patients. The AUC values of OASIS, APACHE II, and SAPS II in predicting 28-day mortality were 0.771 (95% CI [0.742, 0.799]), 0.764 (95% CI [0.735, 0.792]), and 0.767 (95% CI [0.739, 0.796]), respectively, which were higher than that of SOFA (0.686; *p* < .001). Table 3 shows the pairwise comparison of the ROC curves, and there were no statistically significant differences between the AUC values of OASIS, APACHE II, and SAPS II in predicting 28-day mortality.

**Table 3.** Pairwise comparison of ROC curves for predicting 28-day mortality in AKI patients.

Variables	Difference between areas	Standard error	Z	*p*
APACHEII–OASIS	0.00706	0.0115	0.612	.5408
APACHEII–SAPSII	0.00377	0.0112	0.336	.737
APACHEII–SOFA	0.0774	0.0155	4.995	**<.0001**
OASIS–SAPSII	0.00329	0.0125	0.264	.7917
OASIS–SOFA	0.0845	0.0172	4.91	**<.0001**
SAPSII–SOFA	0.0812	0.0167	4.864	**<.0001**

the Oxford Acute Severity of Illness Score; APACHE II: the Acute Physiology and Chronic Health Evaluation II; SOFA: the Sequential Organ Failure Assessment; SAPS II: the Simplified Acute Physiology Score II. Bold values are statistically significant at *p* < .05

### The predictive ability of OASIS, APACHE II, SAPS II, and SOFA score for poor outcomes

The ROC curves for the prediction of 28-day mortality, ICU mortality, and in-hospital mortality by each severity scale are shown in Table 4. The sensitivities, specificities, positive predictive values, and negative predictive values of the optimal cutoff values (from the Youden index) for each scale to predict the three outcomes are listed in Table 5. The cutoff value of OASIS for the prediction of 28-day mortality was 33 with a sensitivity of 87.75% and specificity of 46.26%, as calculated by the ROC curve analysis. OASIS ≥ 33 predicts poor short-term prognosis in patients with AKI.

**Table 4.** Area under the curve of various parameters for predicting poor outcomes in AKI patients.

Severity of illness	AUC	Standard error	*p*	95%confidence interval	
				Lower limit	Upper limit
**28-day mortality**					
OASIS	0.771	0.015	<.001	0.742	0.799
APACHEII	0.764	0.014	<.001	0.735	0.792
SAPSII	0.767	0.015	<.001	0.739	0.796
SOFA	0.686	0.017	<.001	0.653	0.719
**ICU mortality**					
OASIS	0.804	0.014	<.001	0.777	0.832
APACHEII	0.800	0.014	<.001	0.773	0.827
SAPSII	0.801	0.014	<.001	0.774	0.829
SOFA	0.689	0.018	<.001	0.654	0.724
**Hospital mortality**					
OASIS	0.783	0.014	<.001	0.756	0.811
APACHEII	0.776	0.014	<.001	0.748	0.804
SAPSII	0.784	0.014	<.001	0.757	0.811
SOFA	0.676	0.017	<.001	0.643	0.709

AUC: area under the receiver operating characteristic curve; the Oxford Acute Severity of Illness Score; APACHE II: the Acute Physiology and Chronic Health Evaluation II; SOFA: the Sequential Organ Failure Assessment; SAPS II: the Simplified Acute Physiology Score II.

**Table 5.** Performance of multivariable models for predicting poor outcomes in AKI patients.

Severity of illness	YI	Cutoff	Sen%	Spe%	+LR	-LR	PPV	NPV
**28-day mortality**								
APACHEII	0.3976	17	63.02	76.73	2.71	0.48	87.2	45.3
SAPSII	0.4142	39	61.37	80.06	3.08	0.48	88.5	45.2
SOFA	0.2885	7	60.15	68.70	1.92	0.58	82.8	40.7
OASIS	0.4305	33	87.75	46.26	2.55	0.40	86.5	49.6
**ICU mortality**								
APACHEII	0.4562	16	57.95	87.67	4.70	0.48	94.0	38.4
SAPSII	0.4794	39	60.62	87.33	4.78	0.45	94.1	39.9
SOFA	0.2877	7	58.56	70.21	1.97	0.59	86.8	33.7
OASIS	0.4930	33	69.85	79.45	3.40	0.38	91.9	44.1
**Hospital mortality**								
APACHEII	0.4200	16	59.53	82.47	3.40	0.49	89.4	45.2
SAPSII	0.4499	39	62.53	82.47	3.57	0.45	89.8	47.1
SOFA	0.2677	7	59.65	67.12	1.81	0.6	81.8	40.2
OASIS	0.4636	33	71.84	74.52	2.82	0.38	87.4	51.7

YI: Youden’s index; Sen: sensitivity; Spe: specificity; LR+: positive likelihood ratio; LR−: negative likelihood ratio; NPV: negative predictive value; PPV: positive predictive value; the Oxford Acute Severity of Illness Score; APACHE II: the Acute Physiology and Chronic Health Evaluation II; SOFA: the Sequential Organ Failure Assessment; SAPS II: the Simplified Acute Physiology Score II.

### Logistic regression analyses of 28-day mortality in AKI patients

Logistic regression model was used to test the efficacy of OASIS in predicting 28-day mortality in patients with AKI (Table 6). Because OASIS is collinear with APACHE II and SAPS II, variables considered for multivariable analysis included age, sex, OASIS, SOFA, use of vasoactive drugs, MV, RRT, and underlying diseases. OASIS was entered as a continuous variable and a categorical variable (the cutoff value of OASIS was 33), respectively. Multivariable analysis showed that 28-day mortality increased by 8.5% (95% CI, 1.065–1.106) for every point increase in the OASIS, and the 28-day mortality of patients with high OASIS was 3.826 times higher than that of patients with low OASIS. In addition to OASIS, sepsis (OR, 1.823; 95% CI, 1.339–2.481), RRT (OR, 1.802; 95% CI, 1.263–2.570), old age (OR, 1.012; 95% CI, 1.003–1.021), higher SOFA score (OR, 1.091; 95% CI, 1.042–1.141), and MV (OR, 2.016; 95% CI, 1.416–2.871) were significantly associated with a higher risk of death in multivariable analysis.

**Table 6.** Logistic regression analyses of 28-day mortality in AKI patients.

variable	*p*	OR (95 CI%)	Variable	*p*	OR (95 CI%)
Sepsis	<.001	1.823 (1.339–2.481)	Sepsis	<.001	2.002 (1.479–2.711)
RRT	<.001	1.802 (1.263–2.570)	RRT	.002	1.755 (1.232–2.500)
Age	.013	1.012 (1.003–1.021)	Age	.001	1.016 (1.007–1.025)
OASIS^a^	<.001	1.085 (1.065–1.106)	OASIS^b^	<.001	3.826 (2.724–5.326)
SOFA	<.001	1.091 (1.042–1.141)	SOFA	<.001	1.123 (1.075–1.173)
MV	<.001	2.016 (1.416–2.871)	MV	.001	1.779 (1.251–2.529)

Multivariable logistic regression to assess the association of OASIS with 28-day mortality.

^a^OASIS was entered as a continuous variable.

^b^OASIS was entered as a categorical variable, the cutoff value of OASIS was 33.

AKI: acute kidney injury; RRT: renal replacement therapy; the Oxford Acute Severity of Illness Score; SOFA: Sequential Organ Failure Assessment; MV: mechanical ventilation; OR: odds ratio; CI: confidence interval.

### Subgroup analyses

According to the KDIGO criteria, AKI patients were divided into stage 1, stage 2, and stage 3. Patient characteristics by AKI stage are shown in Table S2. The ROC curves of OASIS, APACHE II, SAPS II, and SOFA score for predicting of 28-day mortality in each subgroup are shown in Table 7. OASIS had a good predictive effect in each subgroup. Table 7 shows the calibration of the risk scores. OASIS had good calibration in each subgroup, except the stage 3 subgroup. To verify the predictive effect of OASIS on the 28-day mortality among different populations of AKI patients, we divided the AKI patients into elective surgery, non-elective surgery, MV, non-MV, Sepsis, non-sepsis, RRT, non-RRT, over 65 years, and up to 65 years groups, as shown in Table S3. OASIS had a good prediction effect in most subgroups.

**Table 7.** Receiver operating characteristic curves of risk scores for predicting of 28-day mortality of each subgroup according to KDIGO criteria in AKI patients.

Group	APACHEII	SAPSII	SOFA	OASIS
Stage 1	**0.780^a^**	0.803**^a^**	0.657**^a^**	0.767**^a^**
Stage 2	0.728**^a^**	0.707**^a^**	0.619**^a^**	**0.765^a^**
Stage 3	0.675**^a^**	0.709	0.649**^a^**	**0.730**

AKI: acute kidney injury; AUC: area under the receiver operating characteristic curve; APACHE II: the Acute Physiology and Chronic Health Evaluation II; SAPS II: the Simplified Acute Physiology Score II; SOFA: the Sequential Organ Failure Assessment; the Oxford Acute Severity of Illness Score.

^a^*p* > .05 for Hosmer-Lemeshow test, reflecting good calibration. The risk score column in bold has the highest AUC value for each group.

## Discussion

In this large, multicenter prospective study, we evaluated the ability of the OASIS, APACHE II, SAPS II, and SOFA score to predict the 28-day mortality in AKI patients, and we found that the performance of OASIS was the best, followed by APACHE II and SAPS II, but there were no significant differences among the three scoring systems. The predictive value of the SOFA score was the worst, and the difference was statistically significant compared with the other three scores. OASIS was significantly associated with a higher risk of death in the logistic regression model, whether as a continuous variable or a categorical variable, which further indicated that OASIS had good value in judging the severity of AKI patients. OASIS has been studied in the mixed ICU [20], in the cardiac ICU [21–23], in patients with sepsis [24–28], and in patients admitted to the surgical intensive care unit (SICU) [29], but to date, there has been no study on OASIS in AKI patients.

Deliberato et al. performed a secondary analysis of the electronic health records of patients included in the eICU Collaborative Research Database (eICU-CRD), 108 402 patients in 189 different ICUs across the USA were included in the analysis [20]. In this study, underweight patients had higher OASIS scores, the median value was 31 (IQR: 25, 38), and OASIS demonstrated good discrimination (AUC = 0.79 (0.78–0.80)) in predicting in-hospital mortality in all body mass index (BMI) subgroups. Our study showed similar results, the median value was 31 (IQR: 21, 39), and the performance of OASIS in predicting in-hospital mortality was good (AUC = 0.783 (0.756–0.811)).

Recently, Hu et al. [26] collected the data of 2470 sepsis patients recorded in the Medical Information Mart for Intensive Care III (MIMIC-III) database [30] from 2001 to 2012 and retrieved the SOFA, SAPSII, OASIS and Logistic Organ Dysfunction System (LODS) scores [31] of the patients within the first day admission to the ICU and compared the predictive value of the four scoring systems for ICU mortality of the patients. The AUC values of SAPSII and OASIS were 0.768 (0.745–0.791) and 0.762 (0.738–0.785), respectively, which were significantly higher than those of the other two scoring systems. In our study, the AUC values of OASIS, APACHE II and SAPS II in predicting the 28-day mortality of AKI patients were 0.771 (0.742–0.799), 0.764 (0.735–0.792), and 0.767 (0.739–0.796), respectively, which were higher than that of the SOFA score (0.686; *p* < .001) (Figure 3). Although the subjects of the two studies were different, we both found that OASIS had good predictive value for mortality in ICU patients. In contrast, another study found that SAPS II (AUC = 0.741 (0.703–0.778)) and SOFA score (AUC = 0.687 (0.645–0.728)) showed significantly and slightly better discrimination than OASIS (AUC = 0.684 (0.643–0.725)) [28]. More clinical studies are needed to investigate the validity of OASIS.

In our study, with the increase in OASIS, the mortality rate of patients increased (Figure 2). In AKI patients, OASIS of the non-survivors was higher than that of the survivors (39 vs 28, *p* < .001), which is consistent with the finding of another study (38 vs. 33, *p* < .001) [24], indicating that OASIS has a good predictive value for the 28-day mortality. Moreover, it has a good predictive value for both the ICU mortality and the in-hospital mortality, which is similar to the findings of the original study, hospital and ICU mortality increased exponentially as OASIS increased [14]. In contrast to that in the original study, the AUC value of OASIS in our study (AUC= 0.771) was significantly lower than that of the former (AUC = 0.902), but significantly higher than that of Chen et al. (AUC = 0.652) [24]. The reasons are as follows: first, the original research was performed in a mixed ICU, and the other study was conducted in septic patients, while our subjects were AKI patients. Second, the original research admitted 72 474 ICU patients in 68 ICUs at 49 U.S. hospitals, the other study was conducted using data from a public database, and a total of 10 305 septic patients were included. The large sample size of the studies reduced selection bias and made the results more convincing. Third, the above two studies were retrospective cohort studies, while ours was a prospective observational study. Retrospective studies are prone to confusion and bias.

ROC curve analysis showed that the cutoff value of OASIS for predicting 28-day mortality in AKI patients was 33, OASIS had the highest sensitivity (87.75%) for predicting 28-day mortality, but a lower specificity (46.26%). In another study, the best threshold of OASIS was 34.5, with a specificity of 55.80% and a sensitivity of 64.93% [24]. We divided AKI patients into three subgroups according to the KDIGO criteria, OASIS increased with the increase of the AKI classification (Table S2); Table 7 shows that OASIS had a good predictive value in each subgroup. We also divided AKI patients into groups based on their characteristics. Other studies also grouped subjects to determine the predictive value of OASIS, for example, septic patients were grouped according to age [25], cardiac intensive care unit (CICU) populations were grouped by admission diagnosis [21], and ICU patients were grouped by BMI [20]. Subgroup analysis may be more predictive of patient outcomes. In addition, OASIS includes elective surgery, which is not included in other scores. There were 1459 (49.4%) elective surgery patients in our study, so it may be more meaningful to use OASIS to evaluate their prognosis.

There were some limitations in the present study. First, we did not consider factors such as the etiology, duration, and whether RRT was used for AKI, which might affect the predictive power of OASIS. Second, although this was a prospective study, OASIS was not included in the study design, resulting in incomplete OASIS data for some patients. Third, all AUC values were less than 0.8, indicating that the four risk scores may be inaccurate for AKI patients, which prompt further prospective studies and the development of new scales in this population. OASIS is not widely used at present, probably because it is simpler than APACHE II and SAPS II, but more complex than the SOFA score. Moreover, the values used are all the worst ones selected from the daily minimum and maximum values. If the data records of patients are incomplete, the application of OASIS will be limited. At present, most studies on OASIS are retrospective studies [14,20,22,23,25–29]; therefore, large-scale prospective studies are needed to further verify the predictive value of OASIS.

## Conclusion

Because of the simplicity and effectiveness of OASIS, this study recommends the use of OASIS to evaluate 28-day mortality in patients with AKI admitted to the ICU. OASIS ≥ 33 should be considered an indicator of a negative short-term outcome.

## Supplementary Material

Supplemental Material

## Data Availability

The datasets used and analyzed during the current study are available from the corresponding author on reasonable request.

## Statement

Our manuscript was published as a preprint on 28 June 2021. DOI: https://doi.org/10.21203/rs.3.rs −640541/v1. The website is Research Square. We declare that this manuscript has not been formally published in part or in whole elsewhere, nor has it been considered by any other journal.
