# Supplementary material for: The predictive value of the Oxford Acute Severity of Illness Score for clinical outcomes in patients with acute kidney injury
Source: Ren Fail. 2022 Feb 16;44(1):320–8. doi: 10.1080/0886022X.2022.2027247 (PMC8856098; doi:10.1080/0886022X.2022.2027247)
Supplement: Supplemental Material [file IRNF_A_2027247_SM8447.pdf]

Additional files

Additional file 1:

Members of the Beijing Acute Kidney Injury Trial (BAKIT) workgroup

Yuan Xu (xyuan76@sohu.com), Department of Critical Care Medicine, Beijing Tongren Hospital, Capital Medical University, Beijing 100730, China

Jianxin Zhou (jianxinz@yeah.net), Department of Critical Care Medicine, Beijing Tiantan Hospital affiliated to Capital Medical University, Beijing 100050, China

Ang Li (liang_bjfh68@hotmail.com), Department of Critical Care Medicine, Beijing Friendship Hospital, Capital Medical University, Beijing 100050, China

Jingyuan Liu (liujingyuan.bj@hotmail.com), Department of Critical Care Medicine, Beijing Ditan Hospital , Capital Medical University, Beijing 100015, China

Wenxiong Li (lwx7115@sina.com), Surgical Intensive Care Unit, Beijing Chaoyang Hospital , Capital Medical University, Beijing 100050, China

Wenjin Chen (drchenwenjin@gm) Neurological intensive care unit, Xuanwu Hospital, Capital Medical University, Beijing 100053, China

Jianguo Jia (jiajianguo_1@126.com), Surgical Intensive Care Unit, Xuanwu Hospital, Capital Medical University, Beijing 100053, China

Xi Zhu (xizhuccm@163.com), Department of Critical Care Medicine, Peking University Third Hospital, Beijing 100191, China

Penglin Ma (mapenglin1@163.com), Department of Critical Care Medicine, The 309th Hospital of Chinese People's Liberation Army, Beijing 100094, China

Wei Chen (hanwa@yahoo.com.cn) Department of Critical Care Medicine, Beijing Shijitan Hospital, Capital Medical University, Beijing 100038, China

Dongxin Wang (wangdongxin@hotmail.com), Department of Critical Care Medicine, Peking University First Hospital, Beijing 100034, China

Youzhong An (bjicu@163.com), Department of Critical Care Medicine, Peking University People's Hospital, Beijing 100044, China

Qingyuan Zhan (Zhanqy0915@yahoo.com.cn), Department of Critical Care Medicine, China-Japan Friendship Hospital, Beijing 100029, China

Gang Li (xdysw@163.com), Department of Critical Care Medicine, China-Japan Friendship Hospital, Beijing 100029, China

Haitao Zhang (boy398672@yahoo.cn), Surgical Intensive Care Unit, Fuwai Hospital, China Academy of Medical Science and Peking Union Medical College, Beijing 100037, China

Bo Ning (ningboicu@tom.com), Department of Critical Care Medicine, Air Force General Hospital of Chinese People's Liberation Army, Beijing 100142, China

Zhongjie He (drhezhj@126.com), Department of Critical Care Medicine, The First Affiliated Hospital of General Hospital of People's Liberation Army, Beijing 100048, China

Zhicheng Zhang (zhangzhichengicu@hotmail.com), Department of Critical Care Medicine, Navy General Hospital, Beijing 100048, China

Yaxiong Sun (1073791787@qq.com), Department of Critical Care Medicine, The Luhe Teaching Hospital of the Capital Medical University, Beijing 101149, China

Shijie Jia (jiashj1964@sina.com), Surgical Intensive Care Unit, Beijing Anzhen Hospital, Capital Medical University, Beijing 100029, China

Yalin Liu (icu9999@sina.com.cn), Surgical Intensive Care Unit, Beijing Hospital, Beijing 100005, China

Rui Cheng (chengrui2017@163.com), Department of Critical Care Medicine, General Hospital of Armed Police Forces, Beijing 100039, China

Qing Song (songqing3010301@sina.com), Department of Critical Care Medicine, The General Hospital of People's Liberation Army, Beijing 100039, China

Jinning Liu (jin_ning_liu@163.com), Surgical Intensive Care Unit, Beijing YouAn Hospital, Capital Medical University, Beijing 100069, China

Yangong Chao (chaoyg1059@263.net), Department of Critical Care Medicine, Hua Xin Hospital, First Hospital of Tsinghua University , Beijing 100016, China

Huizhen Li (huizl630@163.com), Department of Critical Care Medicine, Beijing Shunyi Hospital of China Medical University, Beijing 101300, China

Li Feng (bjfengli668@sina.com), Department of Critical Care Medicine, Beijing Geriatric Hospital, Beijing 100095, China

Ruochun Shi (jinxier@163.com), Department of Critical Care Medicine, Beijing No.6 Hospital, Beijing 100007, China

Department of Critical Care Medicine, Fuxing Hospital, Capital Medical University, Beijing 100038, China: Ying Wen (Christina7622@163.com), Qi Jiang (jiangqi7676@sina.com), Peng Wang (438867228@qq.com), Yujie Deng (missydyj@gmail.com), Yan Sun (sunyan198408011717@163.com), Yanyan Yin (yinyanyan678@163.com), Xin Zhang (wood678@sohu.com), Li Zhang (letmedo@sina.com), Zhen Zhao (maggiezhao77@163.com), Ying Wang (butterfly_5643358@hotmail.com), Ran Lou (springin12@sina.com), Jing Wang (monica_jojo2003@yahoo.com.cn)

Additional file 2:

All other ethical bodies that approved our study in the various centers involved.

The institutional review board of Peking Union Medical College Hospital

The institutional review board of Beijing Tongren Hospital, Capital Medical University

The institutional review board of Beijing Tiantan Hospital affiliated to Capital Medical University

The institutional review board of Beijing Friendship Hospital, Capital Medical University

The institutional review board of Beijing Ditan Hospital , Capital Medical University

The institutional review board of Beijing Chaoyang Hospital, Capital Medical University

The institutional review board of Xuanwu Hospital, Capital Medical University

The institutional review board of Peking University Third Hospital

The institutional review board of Peking University First Hospital

The institutional review board of Peking University People’s Hospital

The institutional review board of China-Japan Friendship Hospital

The institutional review board of The 309th Hospital of Chinese People’s Liberation Army

The institutional review board of Beijing Shijitan Hospital, Capital Medical University

The institutional review board of Fuwai Hospital, China Academy of Medical Science and Peking Union Medical College

The institutional review board of Air Force General Hospital of Chinese People’s Liberation Army

The institutional review board of The First Affiliated Hospital of General Hospital of People’s Liberation Army

The institutional review board of Navy General Hospital

The institutional review board of The Luhe Teaching Hospital of the Capital Medical University

The institutional review board of Beijing Anzhen Hospital, Capital Medical University

The institutional review board of Beijing Hospital

The institutional review board of General Hospital of Armed Police Forces

The institutional review board of The General Hospital of People’s Liberation Army

The institutional review board of Beijing YouAn Hospital, Capital Medical University

The institutional review board of HuaXin Hospital, First Hospital of Tsinghua University

The institutional review board of Beijing Shunyi Hospital of China Medical University

The institutional review board of Beijing Geriatric Hospital

The institutional review board of Beijing No.6 Hospital
